# Accuracy and response repeatability of three large language models on undergraduate operative dentistry multiple-choice questions

**DOI:** 10.3389/fdmed.2026.1934573

**Published:** 2026-08-17

**Authors:** Jasmine Mary Antony, Nikhil Harikrishnan, Nishi Jayasheelan

**Affiliations:** Department of Conservative Dentistry and Endodontics, Yenepoya Dental College (Deemed to be University), Mangalore, Karnataka, India

**Keywords:** artificial intelligence, conservative dentistry, dental education, large language models, operative dentistry

## Abstract

**Introduction:**

Artificial intelligence (AI) has emerged as a valuable tool in the field of dentistry to assist healthcare providers in diagnosis, optimize treatment outcomes, support research, and improve education. Furthermore, the generative aspects of AI have emerged as a powerful tool for dental professionals to tackle clinical and academic challenges. However, the reliability of AI in domains such as undergraduate training remains unverified.

**Aim:**

This study aimed to evaluate the accuracy and consistency of three large language models (LLMs) in answering multiple-choice questions on the undergraduate operative dentistry curriculum to determine their reliability as a supplementary learning resource.

**Methods:**

Sixty multiple-choice questions were formulated from the undergraduate operative dentistry curriculum. Each LLM (Claude, Gemini, and ChatGPT) was queried individually nine times over three days. The results were evaluated using a predetermined answer key. The accuracy of the LLMs was compared using a generalized estimating equation (GEE) with question-level clustering analysis. Consistency was assessed at the question level using response consistency, correctness consistency, and Fleiss' kappa. Statistical significance was set at *P* < 0.05.

**Results:**

Gemini demonstrated the highest overall accuracy (98.89%), followed by ChatGPT (98.33%) and Claude (97.78%). No statistically significant difference in accuracy was observed among the three LLMs (GEE, overall Wald *χ*^2^(2) = 1.46, *p* = 0.481). All three models showed almost perfect intersession agreement (Fleiss' *κ* = 0.93–0.95), with response and correctness consistency ranging from 88.3% to 91.7% across models.

**Conclusions:**

All three LLMs demonstrated high accuracy on operative dentistry MCQs, suggesting their potential utility as a supplementary educational tool. However, the residual error rates in the answers warrant the cautious integration of LLMs into dental education.

## 1 Introduction

Generative artificial intelligence (AI) has rapidly become embedded in dental education, used to create quizzes, answer student queries, and provide tailored feedback (1). AI is a subfield of computer science that aims to develop intelligent systems and technologies capable of performing tasks that humans can (2). Equipped with deep learning algorithms, AI chatbots can learn from large amounts of data provided, enhance their performance over time, and adjust to new data and environments (2). The integration of AI into daily life and professional practice has expanded considerably, and with the development of newer architectures and hardware capacities, its potential applications continue to broaden across diverse fields (3). In recent years, there has been a significant increase in the use of AI in dentistry. This technology has been primarily used to diagnose dental diseases, plan treatments, and predict prognosis (4). Furthermore, the generative aspects of AI have emerged as a powerful tool in helping dental professionals tackle clinical and academic challenges (2). These developments have opened new opportunities to support learning in various ways.

At the forefront of these advancements are large language models (LLMs), such as Gemini, ChatGPT, and Claude. Powered by OpenAI, generative pretrained transformer (GPT) is trained on diverse internet texts. By understanding the patterns of the training texts, ChatGPT can improve its responses over time (5). However, the answers are without expert supervision, raising concerns about accuracy in the specialized field. Developed by Google, Gemini leverages the company's expertise in search and language understanding. It employs a transformer-based model that relies on understanding the context of words in a sentence for accurate representation (6). Launched in 2023, Anthropic Claude emphasizes alignment with user needs and excels at concise and reasoned outputs (7).

Previous studies on the application of AI in dental education have yielded contrasting results. A study by Ahmed et al. found that LLMs with an accuracy of 80% significantly outperformed human participants with an average accuracy of 62% in a periodontology in-service examination (8). Meanwhile, Sismanoglu et al. reported a superior performance of human participants as opposed to LLMs in answering the Turkish dental specialization exam (9). Mumcu et al. in 2025 revealed higher accuracy (79%–89%) with LLMs in answering endodontic questions (10). Kavadalla et al. evaluated the implementation of ChatGPT among undergraduate dental students, offering an early picture of how LLMs perform when integrated into coursework (11). However, the accuracy and response reproducibility of different LLMs in operative dentistry have not been evaluated at the undergraduate level. This study aimed to evaluate the accuracy and consistency of three LLMs in answering multiple-choice questions on the undergraduate operative dentistry curriculum to determine their reliability as supplementary learning resources. The null hypothesis states that there is no statistically significant difference in accuracy and consistency among the three LLMs evaluated.

## 2 Materials and methods

### 2.1 Study design

This repeated-query benchmarking study evaluated the performance of three LLMs.

- Google Gemini (Gemini 3 Pro Version)
- ChatGPT (GPT-5.3 version)
- Claude (claude-3.5 sonnet version)

This study was conducted at a dental teaching institute in South India between January 2026 and February 2026. All models were accessed through the free tier between January 31, 2026, and February 2, 2026. As the study did not involve human subjects, ethical approval was waived by the institutional review board.

### 2.2 Questionnaire development

Sixty multiple-choice questions were formulated with reference to the DCI undergraduate operative dentistry curriculum. The questions were developed by a panel of three subject experts in operative dentistry, each with over 10 years of experience and holding the rank of Professor. The questions were distributed across various topics, as follows: fundamentals of cavity preparation (*n* = 10), dental caries (*n* = 8), dental materials (*n* = 10), infection control (*n* = 8), pulp protection (*n* = 8), indirect restorations (*n* = 8), and occlusion (*n* = 8). This ensured adequate coverage of the curriculum. There was a consensus among the authors regarding the clarity of the questions prepared. An answer key was prepared and served as the reference for evaluating the responses generated by the LLMs. (Appendix S1)

### 2.3 Data collection protocol

The LLMs were presented with a series of 60 multiple-choice questions using the same prompt across models. “You will be given a multiple-choice question from the undergraduate operative dentistry curriculum. Select the single best answer from the options provided. Respond with only the option letter and the exact text of the correct option. Do not provide any explanation, reasoning, or additional commentary.” Strict instructions were given to keep the answers short and to avoid explanations. To ensure methodological consistency and control for potential variations in word-context relationships, each question was presented using a standardized copy-and-paste method. The order of the sixty questions was randomized across all testing sessions. No further follow-up questions were asked. All questions were repeated three times a day (morning, noon, and evening) for three consecutive days as a new conversation in a web interface, with the memory feature turned off, to ensure the reliability of the findings. Each response was then evaluated by a single operator who was blinded to the groups. Evaluation was based on the predetermined answer key, which was verified by consensus among the three subject experts.

### 2.4 Statistical analysis

The data were coded in MS Excel, and all statistical analyses were conducted using IBM SPSS 27. The summary of categorical variables is reported as frequencies, percentages, and charts. Accuracy was analyzed using a generalized estimating equation (GEE) with a binomial distribution and logit link, response correctness as the outcome, LLM as the fixed effect, and question identity as the clustering variable. Consistency was assessed at the question level. For each model, the percentage of questions answered identically across all nine sessions and the percentage answered correctly in every session were calculated and inter-session agreement was assessed via Fleiss' kappa.

## 3 Results

**Figure 1:**
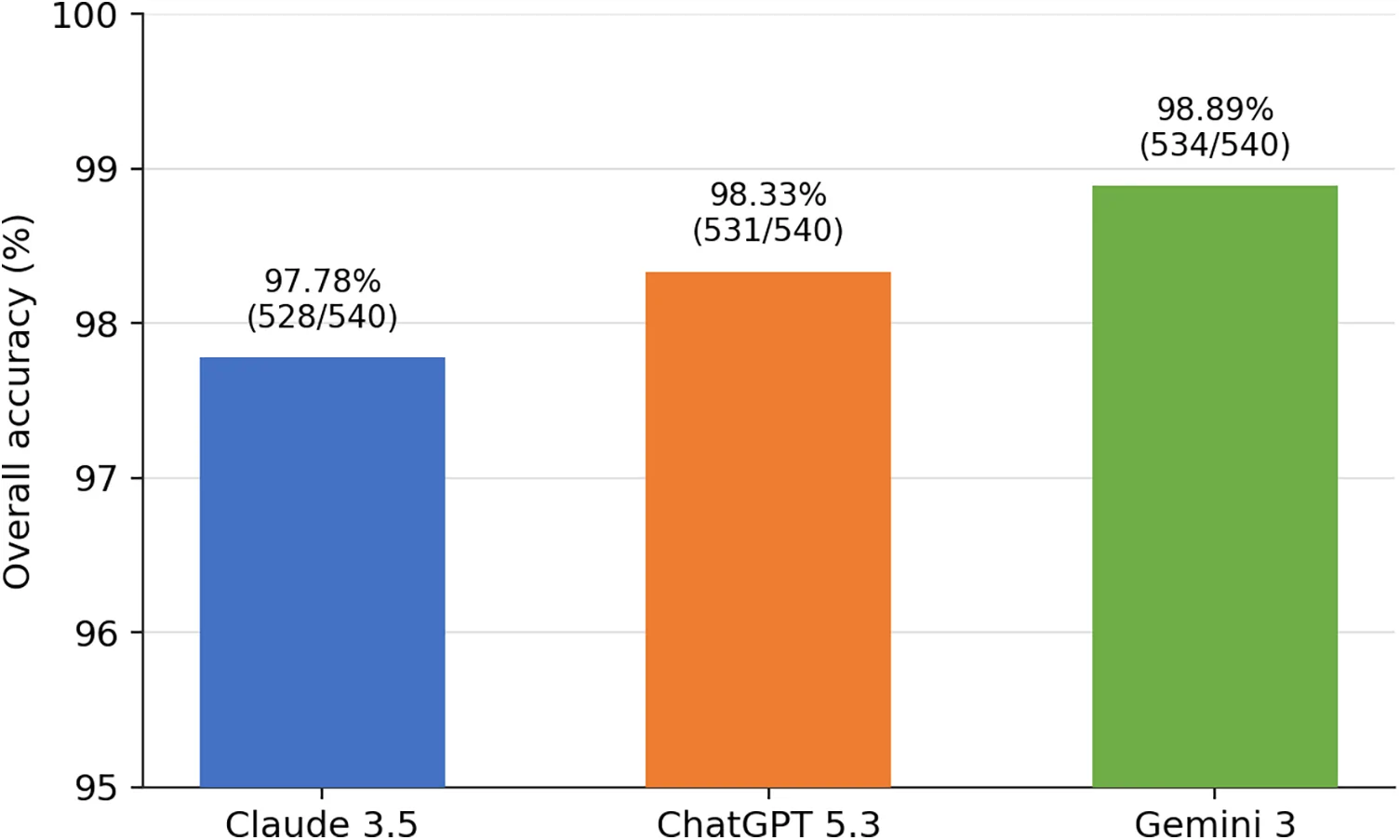
Overall accuracy of each LLM across 540 responses.

A total of 60 questions were asked to three different LLMs (Gemini 3, ChatGPT 5.3, and Claude 3.5) at three different time points over a course of three days. A total of 540 answers were obtained from each LLM. All three LLMs exhibited high accuracy in answering MCQs from the DCI undergraduate curriculum. Claude 3.5 answered 528 out of 540 questions correctly (97.78%). ChatGPT 5.3 answered 531 out of 540 questions correctly (98.33%). Gemini 3 answered 534 out of 540 questions correctly (98.89%) (Figure 1).

Accuracy was compared using a GEE analysis with no statistically significant difference found between the three models (overall Wald *χ*^2^(2) = 1.46, *p* = 0.481). Pairwise comparisons were likewise non-significant: Claude 3.5 vs. ChatGPT 5.3 (OR = 0.75, 95% CI 0.23–2.42, *p* = 0.625), Gemini 3 vs. ChatGPT 5.3 (OR = 1.51, 95% CI 0.62–3.65, *p* = 0.361), and Gemini 3 vs. Claude 3.5 (OR = 2.02, 95% CI 0.58–7.06, *p* = 0.267) (Table 1).

**Table 1:** Pairwise comparison of accuracy between LLMs using a generalised estimating equation (GEE) with binomial family, logit link, and question-level clustering.

Pairwise comparison	Odds Ratio	95% CI	*p*-value
Claude 3.5 vs. ChatGPT 5.3	0.75	0.23–2.42	0.625
Gemini 3 vs. ChatGPT 5.3	1.51	0.62–3.65	0.361
Gemini 3 vs. Claude 3.5	2.02	0.58–7.06	0.267
Overall model effect (Wald *χ*², df = 2)	***χ*^2^** **=** **1.46**	**0.481**	

Bold values indicate the overall model effect.

Claude 3.5 and Gemini 3 each gave an identical answer across all nine sessions for 55 of 60 questions (91.7% response consistency), while ChatGPT 5.3 did so for 53 of 60 questions (88.3%). Inter-session agreement, quantified using Fleiss' kappa, was almost perfect for all three models: Claude 3.5 (*κ* = 0.941), ChatGPT 5.3 (*κ* = 0.931), and Gemini 3 (*κ* = 0.954) (Table 2)

**Table 2:** Question-level response and correctness consistency across nine testing sessions, with fleiss’ kappa for inter-session agreement, for each LLM.

Model	Response consistency (identical answer, all 9 sessions)	Correctness consistency (correct, all 9 sessions)	Fleiss’ *κ* (9 sessions as raters)
Claude 3.5	55/60 (91.7%)	55/60 (91.7%)	0.941
ChatGPT 5.3	53/60 (88.3%)	53/60 (88.3%)	0.931
Gemini 3	55/60 (91.7%)	55/60 (91.7%)	0.954

**Figure 2:**
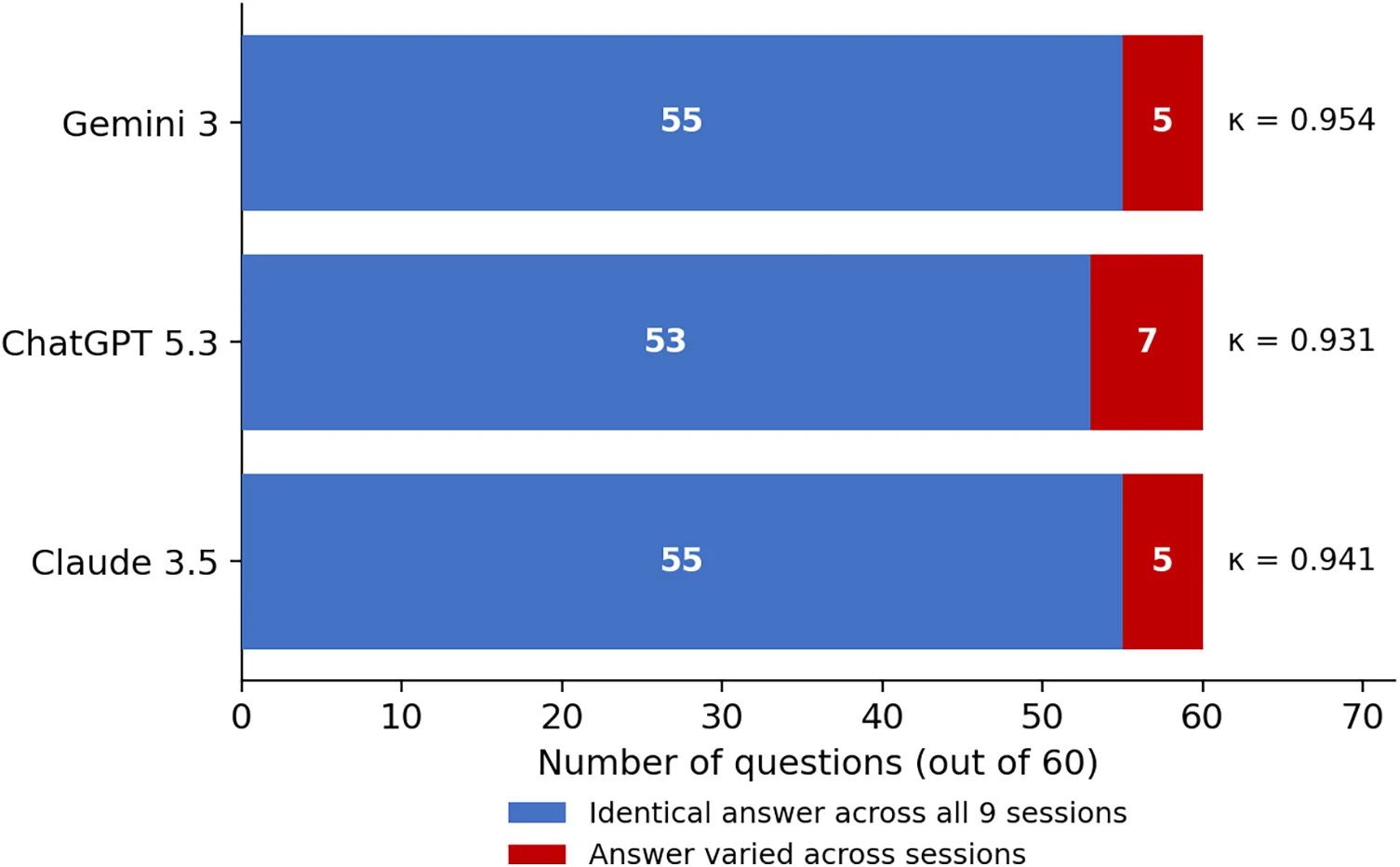
Question-level response consistency across nine sessions with Fleiss’ kappa for inter-session agreement.

Correctness consistency was numerically identical to response consistency for every model (Claude 3.5:55/60, 91.7%; ChatGPT 5.3:53/60, 88.3%; Gemini 3:55/60, 91.7%). In this dataset, inconsistency and inaccuracy were closely linked. A small number of questions accounted for a disproportionate share of the observed variability. Question 1 elicited varying answers from all three LLMs across testing sessions. Claude 3.5 varied on questions 4, 44, 47, and 48; ChatGPT 5.3 varied on questions 4, 7, 11, 13, 15, and 17; and Gemini 3 varied on questions 2, 13, 39, and 43 (Figure 2).

## 4 Discussion

Large language models have become a powerful and easily accessible source of information, with the potential to transform how individuals derive and process information, with a higher impact in healthcare (12). Although LLMs are easy gateways to access medical or dental information, they are often unregulated. It is impossible to police AI, and eliminating the spread of misinformation is an ongoing challenge (13). In dentistry, AI can be utilized for clinical diagnosis, treatment planning, and predicting the prognosis. Their role as a supplementary teaching method warrants academic exploration (14).

In this study, the quality of the information related to the DCI undergraduate operative dentistry curriculum provided by these LLMs, namely, Gemini 3, ChatGPT 5.3, and Claude 3.5, was evaluated in terms of accuracy and consistency. The results showed that all three groups answered with high accuracy, with no significant statistical difference between them. Hence, the null hypothesis was not rejected.

All three evaluated groups performed with high accuracy, with Gemini achieving the highest accuracy among the various groups. This result contrasts with previous studies, where ChatGPT performed significantly better than Gemini (8, 13). Mumcu et al. reported an accuracy ranging from 79% to 89% in answering endodontic questions (10). The present study reported a higher accuracy in answering questions. A possible explanation could be attributed to the nature of the questions evaluated and the question types aligned with the training corpora of the LLMs (15).

Because the 540 responses collected per model represented nine repeated administrations of the same 60 questions rather than independent observations, accuracy was compared using a generalized estimating equation (GEE) with question-level clustering. This analysis found no statistically significant difference in accuracy between the three models. The comparable performance of all three LLMs represents a significant finding. This suggests that the choice of LLMs may not influence the quality of the information provided within this specific domain.

Consistency was assessed at the question level rather than through a comparison of aggregate accuracy across time points. Claude 3.5 and Gemini 3 each gave an identical answer across all nine sessions for 55 of the 60 questions, while ChatGPT 5.3 did so for 53 of 60 questions. Inter-session agreement, quantified using Fleiss' kappa, was almost perfect for all three models. Notably, correctness consistency was numerically identical to response consistency for every model, indicating that no model produced the same incorrect answer consistently across all nine sessions for any question. Every observed error occurred on a question for which the model's answer also varied between sessions. This suggests that, in this dataset, instability in a model's response was closely tied to the underlying difficulty or ambiguity of the question asked, rather than reflecting stable but incorrect knowledge. One question (Q1) elicited a varying answer from all three LLMs, raising the possibility that this question was ambiguously worded or admitted more than one defensible answer, rather than reflecting genuine model instability. Furthermore, the residual errors have a disproportionate risk in an educational domain where the students lack clinical experience to recognize an incorrect or misleading answer.

The answers produced by the three different LLMs across different time points in this study were stable and reproducible. This finding suggests that the information provided was independent of the time of the inquiry. This is in contrast to previous studies, where there was concern regarding the variability of AI-generated responses (16). Kong et al., in a scoping review of LLM applications in dental education, similarly emphasised that performance and stability vary considerably by subject domain, question format, and model version, cautioning against generalising findings across dental specialities or model generation (17).

The limitations of this study are that LLM performance is version-dependent, and performance may change in future software updates and optimizations. This study was conducted within the framework of the DCI undergraduate curriculum, and these findings may not be generalizable to other dental specialties (18). The possibility of data contamination exists because some of the questions were encountered by the LLMs during their pre-training datasets. Future research should explore LLM performance across various formats and education levels, as well as investigate the impact of prompt design and question complexity.

## 5 Conclusion

Within the limitations of this study, all three LLMs demonstrated high accuracy and consistency in responding to DCI undergraduate curriculum MCQs. No statistically significant difference was observed between the groups. All three models also showed high inter-session agreement on the same set of questions. These findings suggest that LLMs hold promise as a supplementary education tool for undergraduate dental students. However, their use should be considered as an adjunct to structured academic instruction and guided learning, rather than a substitute for it, with continued monitoring as models and their capabilities evolve.

## Data Availability

The original contributions presented in the study are included in the article/Supplementary Material, further inquiries can be directed to the corresponding author/s.
