# Supplementary material for: Accuracy and response repeatability of three large language models on undergraduate operative dentistry multiple-choice questions
Source: Front Dent Med. 2026 Aug 17;7:1934573. doi: 10.3389/fdmed.2026.1934573 (PMC13526938; doi:10.3389/fdmed.2026.1934573)
Supplement: Supplementary file 1 [file Table1.docx]

**Conservative Dentistry & Dental Materials — MCQ Questionnaire**

| **Topic** | **No. of Questions** |
| --- | --- |
| Fundamentals of Cavity Preparation | 10 |
| Dental Caries | 8 |
| Dental Materials | 10 |
| Infection Control | 8 |
| Pulp Protection | 8 |
| Indirect Restorations | 8 |
| Occlusion | 8 |
| **Total** | **60** |

**Section A: Fundamentals of Cavity Preparation (Q1–Q10)**

**Q1.** G.V. Black's classification of cavities is primarily based on:

a) Depth of the lesion

b) Site and size of the lesion

c) Location/surface of the tooth involved

d) Etiology of the lesion

**Q2.** A Class II cavity, according to G.V. Black's classification, involves:

a) Pit and fissure of posterior teeth

b) Proximal surfaces of posterior teeth

c) Proximal surfaces of anterior teeth involving incisal angle

d) Cervical third of any tooth

**Q3.** The principle of cavity preparation that refers to the shape given to a cavity to prevent displacement of the restoration under occlusal forces is:

a) Retention form

b) Resistance form

c) Convenience form

d) Outline form

**Q4.** The axio-pulpal line angle is formed in which class of cavity preparation?

a) Class I

b) Class II

c) Class III

d) Class V

**Q5.** "Extension for prevention" as a principle of cavity preparation was given by:

a) Black

b) Markley

c) Sturdevant

d) Ferrier

**Q6.** The internal line angle formed by the junction of the axial wall and the pulpal wall is called: a) Axio-pulpal

b) Dentino-enamel

c) Buccogingival

d) Linguoaxial

**Q7.** Which of the following instruments is primarily used to remove carious dentin during cavity preparation?

a) Enamel hatchet

b) Spoon excavator

c) Ball burnisher

d) Amalgam carver

**Q8.** The convenience form in cavity preparation refers to:

a) Shape given for adequate visibility and accessibility

b) Removal of unsupported enamel

c) Shape that resists occlusal forces

d) Retention of the restorative material

**Q9.** According to modern concepts (minimal intervention dentistry), the current trend in cavity design favors:

a) Wide extension for prevention

b) Conservative, tissue-preserving preparation guided by lesion extent

c) Complete removal of all stained dentin regardless of vitality

d) Standardized cavity shape for all lesions

**Q10.** A "dovetail" is incorporated in the occlusal step of a Class II cavity mainly to provide:

a) Additional resistance form

b) Additional retention form and prevent displacement of proximal portion

c) Better pulp protection

d) Better marginal seal

**Section B: Dental Caries (Q11–Q18)**

**Q11.** The chemico-parasitic theory of dental caries was proposed by:

a) W.D. Miller

b) G.V. Black

c) Keyes

d) Newbrun

**Q12.** The primary microorganism most strongly associated with the initiation of dental caries is: a) Lactobacillus acidophilus

b) Streptococcus mutans

c) Actinomyces viscosus

d) Porphyromonas gingivalis

**Q13.** Keyes' triad for caries etiology includes host, microflora, and:

a) Time

b) Substrate

c) Saliva

d) Fluoride

**Q14.** The zone of dentinal caries characterized by bacterial invasion and irreversible destruction is the:

a) Zone of fatty degeneration

b) Zone of dentinal sclerosis

c) Zone of decomposed dentin (zone of destruction)

d) Zone of translucent dentin

**Q15.** Which dye is commonly used clinically to detect caries-affected/infected dentin during cavity preparation?

a) Methylene blue

b) Caries detector dye

(c) Iodine solution

d) Crystal violet

**Q16.** Radiographically, dental caries typically appears as:

a) A radiopaque area

b) A radiolucent area

c) A mixed radiopaque-radiolucent halo

d) No visible change

**Q17.** The "site and size" classification of caries lesions was proposed by:

a) Black

b) Mount and Hume

c) Markley

d) Pickard

**Q18.** Which of the following is considered a critical pH for enamel demineralization?

a) 7.4

b) 6.5

c) 5.5

d) 4.0

**Section C: Dental Materials (Q19–Q28)**

**Q19.** Which property of a dental material describes its ability to resist indentation or scratching? a) Hardness

b) Ductility

c) Malleability

d) Elasticity

**Q20.** Zinc oxide eugenol (ZOE) cement is primarily used clinically as a:

a) Permanent restorative material

b) Temporary restoration / sedative base

c) Bonding agent

d) Impression material for crown and bridge only

**Q21.** The setting reaction of glass ionomer cement (GIC) is best described as:

a) An acid-base reaction between polyacrylic acid and fluoroaluminosilicate glass

b) A free radical polymerization reaction

c) An exothermic amalgamation reaction

d) A condensation reaction releasing water

**Q22.** In dental amalgam, the primary matrix phase responsible for corrosion and marginal breakdown is:

a) Gamma (γ) phase

b) Gamma-1 (γ1) phase

c) Gamma-2 (γ2) phase

d) Epsilon phase

**Q23.** Which of the following best defines "dimensional stability" of an impression material?

a) Its resistance to tearing

b) Its ability to retain accurate shape/size over time

c) Its flow before setting

d) Its wettability by gypsum

**Q24.** Composite resins primarily set by which mechanism?

a) Acid-base reaction

b) Free radical addition polymerization

c) Amalgamation

d) Hydration reaction

**Q25.** The "working time" of a dental material refers to:

a) Time available to manipulate the material before it starts to set

b) Total time from mixing to final set

c) Time required for the material to reach maximum strength

d) Time taken for material to be removed from the mouth

**Q26.** Which cement has the highest compressive strength and is traditionally used for cementation of cast restorations, though it has largely been replaced?

a) Zinc oxide eugenol cement

b) Zinc phosphate cement

c) Calcium hydroxide cement

d) Polycarboxylate cement

**Q27.** Coefficient of thermal expansion of a restorative material should ideally be:

a) Much higher than tooth structure

b) Much lower than tooth structure

c) Close/similar to that of tooth structure

d) Irrelevant to the restoration's success

**Q28.** Silver diamine fluoride (SDF) is primarily used in current clinical practice for:

a) Cavity liner

b) Arresting/managing dental caries non-invasively

c) Permanent restoration

d) Bleaching agent

**Section D: Infection Control (Q29–Q36)**

**Q30.** The most effective and widely recommended method of sterilization in a dental clinic is:

a) Boiling water

b) Chemical (cold) sterilization

c) Autoclaving (steam under pressure)

d) UV light exposure

**Q29.** "Standard precautions" in infection control imply that:

a) Only patients with known infections require barrier protection

b) All patients are treated as potentially infectious

c) Only surgical procedures require sterile technique

d) Precautions are needed only for HIV-positive patients

**Q31.** The standard autoclave sterilization cycle typically requires steam at 121°C for:

a) 5 minutes

b) 15–20 minutes

c) 60 minutes

d) 2 hours

**Q32.** Biomedical waste in a dental clinic, such as extracted teeth and sharps, should be segregated and disposed of according to:

a) Regular municipal waste guidelines

b) Biomedical Waste Management Rules

c) Personal discretion of the dentist

d) There is no specific requirement

**Q33.** Which of the following is an example of a semi-critical item requiring high-level disinfection or sterilization?

a) Dental Loupes

b) Impression material tray

c) Light-cure gun handle

d) Bracket table surface

**Q34.** The most important single measure to prevent cross-infection between patients in a dental operatory is:

a) Use of mouthwash by the patient

b) Hand hygiene / handwashing by the operator

c) Use of air conditioning

d) Use of scented disinfectants

**Q35.** Personal protective equipment (PPE) in dental practice includes all of the following EXCEPT: a) Gloves

b) Mask

c) Protective eyewear

d) Regular street clothing

**Q36.** Chemical disinfectants such as sodium hypochlorite are commonly used in dental clinics for:

a) Sterilizing handpieces

b) Surface disinfection

c) Root canal obturation

d) Cavity lining

**Section E: Pulp Protection (Q37–Q44)**

**Q37.** The primary purpose of a cavity varnish is to:

a) Provide thermal insulation

b) Reduce microleakage by sealing dentinal tubules

c) Provide mechanical strength to the restoration

d) Neutralize acids from cement

**Q38.** Calcium hydroxide, when used as a pulp-capping agent, promotes:

a) Formation of reparative dentin

b) Dissolution of dentin

c) Permanent obturation of the pulp chamber

d) Bleaching of the tooth

**Q39.** Indirect pulp capping is indicated when:

a) There is frank pulp exposure with purulent discharge

b) Deep caries is close to the pulp but pulp is not exposed and vital

c) The tooth is grossly decayed with no restorable structure

d) The patient has irreversible pulpitis

**Q40.** Direct pulp capping is most likely to succeed when the exposure is:

a) Large, carious, and contaminated

b) Small, mechanical, and in a tooth with a healthy pulp

c) In a tooth with symptoms of irreversible pulpitis

d) In a non-vital tooth

**Q41.** Which material is considered the "gold standard" for pulp capping and pulpotomy due to its biocompatibility and sealing ability?

a) Mineral trioxide aggregate (MTA)

b) Zinc phosphate cement

c) Amalgam

d) Composite resin

**Q42.** The primary role of a cavity liner (e.g., calcium hydroxide liner) placed in a deep cavity is to: a) Provide bulk and resistance form

b) Stimulate reparative dentin formation and protect the pulp

c) Bond the restoration to enamel

d) Provide esthetics

**Q43.** The "smear layer" produced during cavity preparation consists of:

a) Only bacterial debris

b) A layer of debris comprising cut dentin, odontoblastic processes, and bacteria

c) Only saliva contamination

d) Only enamel rods

**Q44.** A cavity base differs from a liner mainly in that a base:

a) Is thinner and used only for sealing dentinal tubules

b) Is thicker and provides mechanical/thermal protection along with some bulk

c) Is used only in enamel

d) Has no protective function

**Section F: Indirect Restorations (Q45–Q52)**

**Q45.** An inlay is best defined as an indirect restoration that:

a) Covers one or more cusps

b) Fits within the confines of the tooth, restoring proximal and occlusal surfaces without cuspal coverage

c) Covers the entire clinical crown

d) Is placed directly in the cavity and cured in the mouth

**Q46.** An onlay differs from an inlay mainly by:

a) Being made of a different material

b) Providing coverage of one or more cusps in addition to proximal/occlusal surfaces

c) Being cemented with a different luting agent

d) Requiring no tooth preparation

**Q47.** The ideal taper (convergence angle) recommended for tooth preparation for a full crown to ensure adequate retention is approximately:

a) 0–2 degrees

b) 6–10 degrees

c) 20–25 degrees

d) 30–40 degrees

**Q48.** A provisional (temporary) restoration during fabrication of an indirect restoration primarily serves to:

a) Provide permanent esthetics

b) Protect the prepared tooth, maintain position, and provide function until the final restoration is cemented

c) Replace the need for a final impression

d) Serve as the definitive restoration if patient is satisfied

**Q49.** Elastomeric impression materials (e.g., addition silicone) are preferred for indirect restorations mainly because of their:

a) Poor dimensional stability

b) Excellent accuracy and dimensional stability

c) Low cost only

d) Inability to reproduce fine detail

**Q50.** The final cementation step of an indirect restoration (e.g., crown) commonly uses which type of cement/material?

a) Zinc oxide eugenol only

b) Resin cement, glass ionomer, or zinc phosphate depending on case

c) Composite flowable resin only

d) Amalgam

**Q51.** A "ferrule" in the context of indirect restorations (crowns) refers to:

a) The impression coping

b) A band of remaining tooth structure encircled by the crown margin for retention and resistance

c) The cement used for luting

d) The metal core buildup only

**Q52.** For posterior teeth, the most common indirect restorative material chosen for its strength and wear resistance is:

a) Composite resin only

b) All-ceramic or metal-ceramic/full-cast restorations

c) Glass ionomer only

d) Compomer only

**Section G: Occlusion (Q53–Q60)**

**Q53.** Centric relation is best defined as:

a) The habitual bite position of the patient

b) A bone-to-bone relationship independent of tooth contact

c) The position of maximum intercuspation

d) The position during mastication only

**Q54.** Centric occlusion refers to:

a) The relationship of the condyle to the fossa

b) The occlusion of teeth when the mandible is in centric relation

c) The maximum intercuspation of teeth, regardless of condylar position

d) The resting position of the mandible

**Q55.** During lateral excursive movements, when the posterior teeth on the working side disclude all other teeth except canines, this is called:

a) Group function

b) Canine guidance

c) Balanced occlusion

d) Cross-bite

**Q56.** An articulator is a device used to:

a) Take impressions

b) Simulate mandibular movements and jaw relationships outside the mouth

c) Measure occlusal forces

d) Sterilize dental instruments

**Q57.** The record used to transfer the relationship of the maxillary arch to the opening axis of the temporomandibular joint (TMJ) to an articulator is called:

a) Bite registration

b) Facebow record

c) Interocclusal record

d) Study cast

**Q58.** In normal occlusion, the mesiolingual cusp of the maxillary first molar occludes with which structure on the mandibular first molar?

a) Buccal cusp

b) Central fossa

c) Marginal ridge only

d) Distolingual cusp tip

**Q59.** "Disclusion" refers to:

a) Complete loss of all teeth

b) Separation of posterior teeth during protrusive/lateral mandibular movements

c) Maximum intercuspation of teeth

d) A type of malocclusion

**Q60.** Freeway space (interocclusal rest space) is the distance between occlusal surfaces of teeth when the mandible is in:

a) Centric occlusion

b) Physiologic rest position

c) Protrusive position

d) Maximum lateral excursion

**Answer Key**

| **Q** | **Ans** | **Q** | **Ans** | **Q** | **Ans** | **Q** | **Ans** |
| --- | --- | --- | --- | --- | --- | --- | --- |
| 1 | c | 16 | b | 31 | b | 46 | b |
| 2 | b | 17 | b | 32 | b | 47 | b |
| 3 | b | 18 | c | 33 | b | 48 | b |
| 4 | b | 19 | a | 34 | b | 49 | b |
| 5 | a | 20 | b | 35 | d | 50 | b |
| 6 | a | 21 | a | 36 | b | 51 | b |
| 7 | b | 22 | c | 37 | b | 52 | b |
| 8 | a | 23 | b | 38 | a | 53 | b |
| 9 | b | 24 | b | 39 | b | 54 | c |
| 10 | b | 25 | a | 40 | b | 55 | b |
| 11 | a | 26 | b | 41 | a | 56 | b |
| 12 | b | 27 | c | 42 | b | 57 | b |
| 13 | b | 28 | b | 43 | b | 58 | b |
| 14 | c | 29 | b | 44 | b | 59 | b |
| 15 | b | 30 | c | 45 | b | 60 | b |
